# Pain in the Ear

**Published:** 1898-03-12

**Authors:** Macleod Yearsley

**Affiliations:** Assistant-Surgeon the Royal Ear Hospital, Surgeon in Charge of the Throat, Nose, and Ear Department, the Farringdon General Dispensary; formerly Clinical Assistant and House Surgeon to the Royal Ear Hospital, &c.


### Medical Progress and Hospital Clinics.

[*The Editor will be glad to receive offers of co-operation and contributions from members of the profession. All letters should be addressed to* The Editor, at the Office, 28 & 29, Southampton Street, Strand, London, W.C.]

### PAIN IN THE EAR.

By Macleod Yearsley, F.R.C.S., Assistant-Surgeon the Royal Ear Hospital, Surgeon in Charge of the Throat, Nose, and Ear Department, the Farringdon General Dispensary; formerly Clinical Assistant and House Surgeon to the Royal Ear Hospital, &c.

### II.—Its Treatment.

Having discussed the diagnosis of pain in the ear the question of its treatment may be proceeded with. In so doing, it is unnecessary to go into the details of operative procedures in cerebral and cerebellar abscess, thrombosis of the lateral sinus, or similar complications, since, unless in very exceptional cases, the treatment of those conditions will hardly be undertaken by the general practitioner. To him their early and accurate diagnosis is of more importance; and the treatment, which is of greater moment, is that of the conditions discussed in the earlier part of the first article.

What has been said of the causes of reflex otalgias gives an indication of their appropriate treatment. The existing trouble—a carious tooth, disease of the tongue, pharynx, or fauces—must be sought for and removed; and, at the same time, general treatment directed against any predisposing cause must be entered upon.

The pain arising from diffuse external otitis can be combatted by the application of cold by means of the ice-bag or Leiter's tubes. Local depletion by leeches is also a valuable means, and, as this will be mentioned more than once, the proper method of applying them to the ears may be at once dealt with. Before using leeches, the external meatus should always be well plugged with cotton wool. This done, the leech should be applied just in front of the tragus, that being the spot where the veins conveying blood from the ear emerge. From one to five leeches may be employed or Huerteloup's artificial leech may be used.

Another useful agent is heat, which may be applied by means of hot, dry wool, or by the instillation of hot sterilised water. Occasionally a similar application of sedatives is of benefit, useful prescriptions being: "℞. Morphinæ hydrochloratis, gr. i; aquæ destill., ℥ ss. Sig. Ten drops to be used hot." And, "℞. Cocainæ hydrochloratis, gr. v.; morphinæ hydrochloratis, gr. i.; aquæ destill., ℥ ss." Sedatives may also be applied by means of "aural ovoids," in which the drug is introduced in gelatine, which slowly dissolves when placed in the meatus. Counter-irritation over the mastoid by liquor epispasticus is of value when the condition is chronic. At times, when there is much swelling, incisions in the meatal wall become necessary.

The pain occasioned by circumscribed otitis externa is, as has been said, of an agonising character, and requires immediate relief. This can only be obtained by incising the boils, a proceeding which must be insisted upon. Incision is very painful, and requires either a local anæsthetic or nitrous oxide gas. Cocain or eucain will suffice, and I have in two cases* found the latter satisfactory. A 1 in 10 solution of eucain hydrochloride (best made fresh by dissolving a five-grain "soloid" in 55 minims of sterilised water) is instilled warm into the external meatus, and allowed to remain for ten minutes, the head being inclined to the opposite side. The meatus is then gently syringed by a 1 in 2,000 solution of hydrarg. perchlor., and the furuncle is opened under a good illumination. This can be done with a short, straight knife, the patient's head being steadied by an assistant. Accidents can be better avoided, however, by using the sharp, hook-shaped knife devised by Dr. Dundas Grant. Incision may be followed by the instillation at intervals of warm glycerine of carbolic acid, which can be used by the patient himself. General treatment is always required. Patients suffering from aural furuncle are generally "run down" and, the pain being always worse at night, require sedatives as well as tonics. The practitioner must not rest satisfied by relieving his patient of one crop of boils, but must endeavour to prevent subsequent attacks. To attain this end attention should be directed to his surroundings, and the influence of sewer gas in causing the trouble (as pointed out by Mr. Field) must be kept in mind. Among preventive drugs, calcium sulphide in doses of gr. $\frac{1}{10}$ to $\frac{1}{4}$ three times a day is recommended. I have found small doses of arsenic useful for the same purpose.

Passing now to pain caused by disease of the middle ear, attention is turned to the treatment of acute catarrhal otitis media. This is both general and local. The ears should be protected from cold by means of wool, and the patient confined to the house, and, if necessary, to his room. The nasal passages and pharynx must be looked to, and the former kept clean by gentle syringing with a warm alkaline wash. The patient should be warned against the unnecessary habit of violently blowing the nose. The bowels should be freely opened, and a diaphoretic mixture ordered. Gentle Politzerisation may be practised provided the nose and nasopharynx are free of foul mucous and discharge; and to ensure this the air douche had better be used only after the nose has been syringed. Inhalation of a medicated vapour is often useful, such as tinct. benzoin. co., oleum pini sylvestris, or thymol. These inhalations should be employed for ten minutes at a sitting, and the patient ordered to inflate the ears by Valsalva's method twice on each occasion. The chloride of ammonium inhaler used in the same manner is also useful. If the pain is very great relief may be obtained by leeching as already described. At all times care must be taken not to allow the condition to pass into the suppurative form, and any bulging of the membrane from retained secretion promptly met by paracentesis. Subsequent attacks should be guarded against by attention to the nose and nasopharynx and by the avoidance of further "colds."

In acute myringitis every vibration of the congested

* *British Medical Journal*, Jan. 16, 1897.

and sensitive membrane intensifies the pain, and the ear must be protected from sound by a pad. Leeches should be applied in front of the tragus, and hot, dry wool or flannel to cover the ear. Instillations are not usually well borne, the weight of the column of fluid being too much for the membrane. General treatment must not be neglected. Politzerisation should be avoided.

In the early stage of acute suppurative otitis media leeches should be promptly applied, in conjunction with dry heat. Instillations of cocaine and morphine, or morphine and atropine, are useful. When the membrane bulges, or there is the least suspicion of pus in the tympanum, paracentesis must on no account be delayed; indeed, its performance by an unpractised hand is preferable to its neglect. The same precautions given in discussing the treatment of furuncle for anæsthetising and antisepticising should first be carried out, and the incision done at the point of bulging by means of a shouldered paracentesis knife. This requires a good illumination and the patient's head to be carefully steadied. The opinion of most otologists now is that this should not be followed by Politzerisation or by syringing. To do so is to expose the patient to the risk of secondarily infecting the cavity of the tympanum, and, worse still, the antrum. After paracentesis, a drain of sterilised gauze should be introduced into the meatus and covered by a gauze dressing in the concha. The drain should be changed in twenty-four hours. Paracentesis alone is in most cases sufficient to relieve the condition, but should pain continue it may become necessary to wash out the tympanum, viâ the Eustachian catheter, with a warm solution of boric acid. If pain persist after this proceeding some complication is almost certainly present.

In treating acute suppuration in the tympanum, the attention must not be confined to the ear, but the nose and pharynx must receive equal care, as must the general condition of the patient himself. And here a few words as to the prevention of suppurative otitis media occurring during the course of specific fevers will not be out of place. Two-fifths of the cases of middle ear disease are due to measles or scarlet fever, and yet such a complication in the course of those diseases is perfectly preventable. Proper attention to the throat and nose during the course of the fever, the gentle use of the handkerchief, the alkaline nasal wash, or nasopharyngeal lavage, will do much towards the prevention of Eustachian blocking, and gentle Politzerisation (with the precautions mentioned above) will keep the tympanum ventilated. In the exanthemata, too, much subsequent suffering may be prevented by timely paracentesis of the membrane.

As already said, the treatment of those grave complications which give rise to pain about the ear will not be entered upon, nor need operations upon the mastoid be discussed. I would rather end this article with a few remarks upon the *prevention* of mastoid empyema, a question of some moment to those suffering from acute or chronic middle-ear suppuration. To think that mastoid empyema is a necessary consequence of otitis media is a mistake, and one cannot but be struck by the influence that a wider knowledge of otology has of late years had in diminishing the number of cases requiring mastoid operations. Probably in every case of acute middle-ear suppuration the mastoid antrum (which must, as pointed out by Cheatle, be looked upon as a part of the tympanum) is involved. This tends to get well with the disease of the rest of the tympanum, and, therefore, the avoidance of Politzerisation or syringing after paracentesis is important, in order to prevent secondary infection and consequent acute mastoid empyema. By preventing the acute form, a large number of chronic empyemata will be also prevented. In dealing with chronic cases, it is useless to attempt the relief of the mastoid complication without primarily effecting a proper treatment of the tympanum, and it is in this that so many general surgeons (who think that otology simply consists in clearing out mastoids or syringing for wax) err, in that they too often open and treat the mastoid process without removing thoroughly the sources of infection in the tympanum.

